# A novel radiomics based on multi-parametric magnetic resonance imaging for predicting Ki-67 expression in rectal cancer: a multicenter study

**DOI:** 10.1186/s12880-023-01123-1

**Published:** 2023-10-27

**Authors:** Xiuzhen Yao, Weiqun Ao, Xiandi Zhu, Shuyuan Tian, Xiaoyu Han, Jinwen Hu, Wenjie Xu, Guoqun Mao, Shuitang Deng

**Affiliations:** 1 Department of Ultrasound, Putuo People’s Hospital, School of Medicine, Tongji University, Shanghai, China; 2 Department of Radiology, Tongde Hospital of Zhejiang Province, No. 234 Gucui Road, Hangzhou, Zhejiang Province 310012 China; 3 Department of Ultrasound, Tongde Hospital of Zhejiang Province, Hangzhou, China; 4 Department of Pathology, Tongde Hospital of Zhejiang Province, Hangzhou, Zhejiang Province China; 5 Department of Radiology, Putuo People’s Hospital, School of Medicine, Tongji University, Shanghai, China; 6 Zhejiang Chinese Medical University, Hangzhou, Zhejiang Province China

**Keywords:** Rectal cancer, Multi-parametric, Radiomics, Ki-67, Magnetic resonance imaging

## Abstract

**Background:**

To explore the value of multiparametric MRI markers for preoperative prediction of Ki-67 expression among patients with rectal cancer.

**Methods:**

Data from 259 patients with postoperative pathological confirmation of rectal adenocarcinoma who had received enhanced MRI and Ki-67 detection was divided into 4 cohorts: training (139 cases), internal validation (in-valid, 60 cases), and external validation (ex-valid, 60 cases) cohorts. The patients were divided into low and high Ki-67 expression groups. In the training cohort, DWI, T2WI, and contrast enhancement T1WI (CE-T1) sequence radiomics features were extracted from MRI images. Radiomics marker scores and regression coefficient were then calculated for data fitting to construct a radscore model. Subsequently, clinical features with statistical significance were selected to construct a combined model for preoperative individualized prediction of rectal cancer Ki-67 expression. The models were internally and externally validated, and the AUC of each model was calculated. Calibration and decision curves were used to evaluate the clinical practicality of nomograms.

**Results:**

Three models for predicting rectal cancer Ki-67 expression were constructed. The AUC and Delong test results revealed that the combined model had better prediction performance than other models in three chohrts. A decision curve analysis revealed that the nomogram based on the combined model had relatively good clinical performance, which can be an intuitive prediction tool for clinicians.

**Conclusion:**

The multiparametric MRI radiomics model can provide a noninvasive and accurate auxiliary tool for preoperative evaluation of Ki-67 expression in patients with rectal cancer and can support clinical decision-making.

## Background

Rectal cancer is a common malignant tumor globally. Its high incidence and mortality threaten patients’ health and quality of life [1]. Locally advanced rectal cancer is generally treated with multimodal strategies involving neoadjuvant chemotherapy, radiotherapy and total mesorectal excision to improve patients’ survival rates. However, data from over the past decade demonstrated that locally advanced rectal cancer had high metastasis rates (29–39%) [2]. Although postoperative adjuvant chemotherapy was adopted, the metastasis rates were greater than twice the rate of primary tumor recurrence [2, 3]. The heterogeneity of molecular pathological characteristics between and inside tumors resulted in different clinical prognosis outcomes [4]; thus, accurate prognostic markers are necessary to facilitate and optimize therapeutic decision-making [5]. Rectal cancer marker detection can indicate the biological characteristics of tumors, which benefits the evaluation of therapeutic efficacy and patient prognosis [6–8].

Among tumor markers, Ki-67 expression is key for the diagnosis of rectal cancer. Ki-67 is a monoclonal antibody for identifying relevant proliferating cell nuclear antigens. Research has indicated that the Ki-67 expression levels are closely related to differentiation levels, infiltration, metastasis, and prognosis of rectal cancer and directly affect prognosis outcomes [9, 10]. However, Ki-67 expression can only be determined through invasive biopsy or surgical pathology tissues [11]. Preoperative examination approaches assessing Ki-67 expression enabling to predict patient prognosis without having to undergo invasive examinations or surgery can thus be beneficial for patients.

MRI is fundamental for preoperative diagnosis in cancer staging, evaluation of therapeutic efficacy, and postoperative follow-up [12–14]; it facilitates comprehensive evaluation of multiple crucial prognostic factors in rectal cancer. Clinically, doctors evaluate tumor characteristics with the imaging features of the lesions. However, this method relies on doctor experience and specialties, lacking repeatability [15, 16]. Therefore, a noninvasive imaging biomarker to predict ki-67 status prior to surgery would offer additional prognostic value and allow more individualized management of patients with rectal cancer.

Radiomics uses high-throughput quantitative image analysis to represent tumors and the relevant microenvironment and can identify more features than visual inspection [16, 17]. Radiomics has extensive applications in rectal cancer research, including: (1) prediction of the therapeutic efficacy of neoadjuvant methods [18]; (2) evaluation of tumor, node, metastasis (TNM) staging [19, 20] and neurovascular invasion [21]; (3) analysis of survival gains after clinical treatment [22]. The performance of established prediction models is varied. Radiomics has been conducted to evaluate Ki-67 expression as a tumor prognostic indicator in breast cancer [23], bladder cancer [24], and gastrointestinal stromal tumors [25, 26]. However, studies have mostly used single-scanning sequences or -modality evaluation methods [25, 26]. Such evaluations have been rarely reported in rectal cancer.

In this study, we developed a multi-parametric MRI radiomics for preoperative prediction of Ki-67 expression in patients with rectal cancer. Clinical data were also used to construct a combined model. The stability and reliability of the model were validated using internal and external data from two centers.

## Methods

### Participants

All experimental protocols were approved by the ethics committees of Tongde Hospital of Zhejiang Province (Center 1, No.TD2021-96) and Shanghai Putuo District People’s Hospital (Center 2, No.PT2022-2). The need for informed consent was waived by the ethics committees of Tongde Hospital of Zhejiang Province and Shanghai Putuo District People’s Hospital to this retrospective study design. All of the procedures were performed in accordance with the Declaration of Helsinki and relevant policies in China. From January 2015 to August 2021, a total of 259 patients with rectum adenocarcinoma were included as participants (163 males and 96 females). The average age was 65.0 ± 11.6 years. The participants were divided into training (139 cases from Center 1), internal validation (in-valid, 60 cases from Center 1), and external validation (ex-valid, 60 cases from Center 2) cohorts. The 199 patients in Center 1 were randomly distributed into the training cohort and the in-valid cohort with a ratio of 7:3. The model was constructed based on the training cohort. Internal and external data validation were conducted to verify the reliability of the model.

**Fig. 1:**
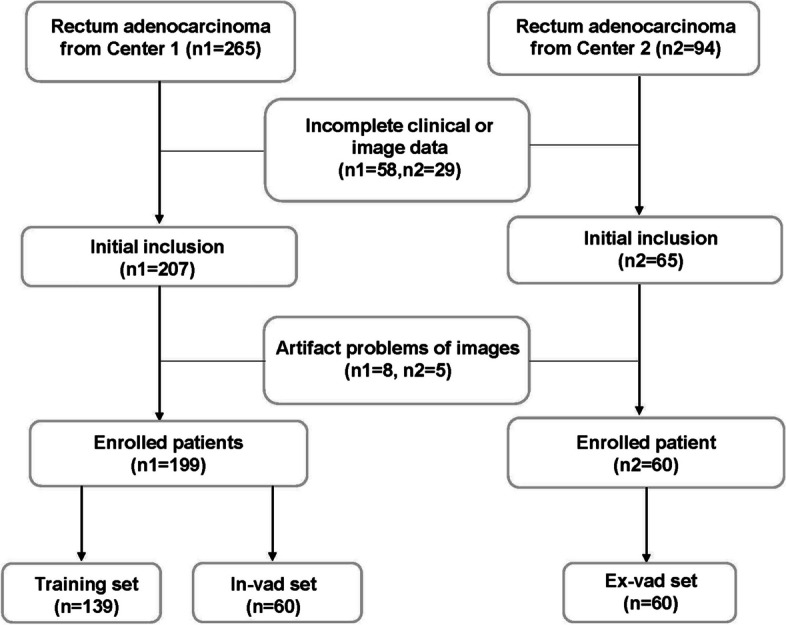
Flow diagram of enrolled patients in this two-center study

The patient inclusion criteria were as follows: (1) The patient was confirmed with rectum adenocarcinoma by surgical pathology and had received immunohistochemistry (IHC) tests for Ki-67. (2) The patient received rectal MRI scanning with contrast enhancement within 2 weeks before surgery. (3) Complete images, clinical data, surgical data, and pathological data were available. The exclusion criteria were as follows: (1) The patient had not received surgery or pathologically confirmed not to be rectum adenocarcinoma. (2) The clinical and image data were incomplete. (3) MRI images had severe artifact problems leading to difficulty in interpreting the images. A flow diagram of patient recruitment is shown in Fig. 1.

### MRI protocol and image analysis

MRI scanning was performed with SIEMENS 3.0T Verio and SIEMENS 1.5T Avanto (Siemens, Germany). The selected sequences included the conventional axial T2WI, DWI, and contrast enhancement T1WI (CE- T1). MRI scanners and scanning Parameters were summarized in Table 1.

**Table 1:** MRI scanning parameters in two centers

Sequence	Parameters	SIEMENS 3.0T Verio	Siemens 1.5T Avanto
T2WI	TR/TE,ms	3900/80	5690/84
	FOV,mm	250 × 380	226 × 250
	Thickness,mm	3	5
	Matrix	256 × 256	512 × 640
DWI	b value	0, 800, 1500	50, 400, 800
	TR/TE,ms	9700/93	4912/95
	FOV,mm	250 × 250	250 × 250
	Thickness,mm	3	5
	Matrix	96 × 160	144 × 192
CE-T1	TR/TE,ms	5.1/1.7	677/12
	FOV,mm	260 × 250	230 × 250
	Thickness,mm	3	5
	Matrix	138 × 192	189 × 256

Prior to contrast-enhanced MRI scanning, the contrast agent Gadopentetate Dimeglumine (Beilu Pharmaceutical Co., China) was injected at a rate of 2.5-3.5ml/s with a dosage of 0.1mmol/Kg through the dorsal metacarpal veins, followed by rinsing with 20 ml saline. The late arterial phase images were selected for processing, when tumors are more strongly enhanced and having good contrast with the surrounding tissues.

**Fig. 2:**
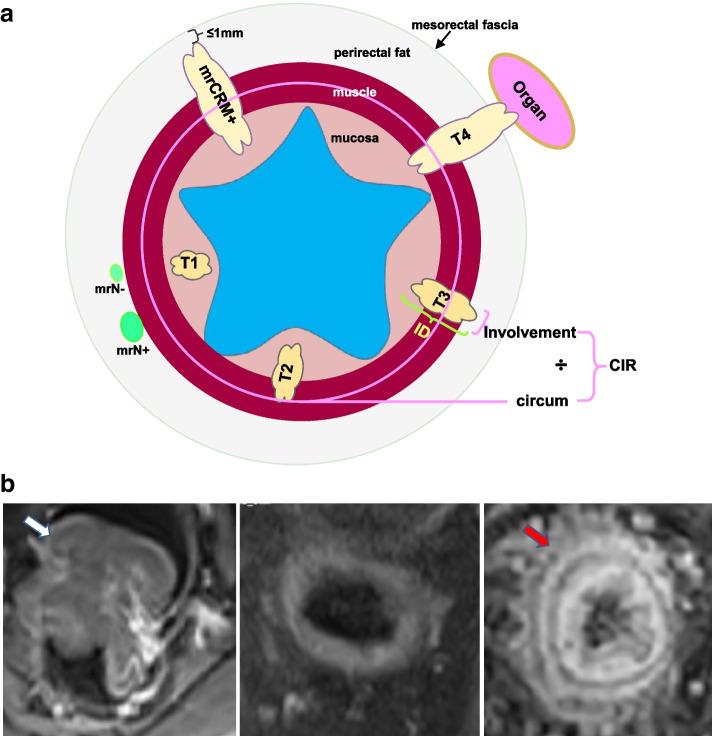
Sketch map of infiltration depth (ID), circum-involvement ratio (CIR), MRI-detected circumferential resection margin (mrCRM), MRI-detected T (mrT) and MRI-detected lymph node (mrN) (**a**) and enhancement pattern (EP) of rectal cancer (**b**). Mild/Moderate (left, white arrow) or obvious (right, red arrow) of tumor compared to normal rectal wall enhancement (middle)

MRI images were viewed by two radiologists with 8 and 15 years of experience in abdominal diagnosis, respectively. The radiologists were not informed of the patients’ histopathological results. They observed the images, identified lesions, and made a diagnosis at a picture archiving and communication system workstation. They reported the following contents: (1) patients’ basic information (Sex、Age、Serum CEA level); (2) neoadjuvant chemotherapy; (3) tumor location (upper, middle, low) ; (4) apparent diffusion coefficient (ADC) values: place region of interest (ROI) at the maximum level of the tumor in the ADC image, with a circular shape that covers the tumor as much as possible; (5) long diameter: tumor length measured on sagittal T2WI; (6) infiltration depth: infiltration depth of tumor measured on axial T2WI; (7) circum-involvement ratio (CIR): the percentage of largest circumferential tumor invasion of the rectal wall; (8) MRI-detected circumferential resection margin (mrCRM): positive mrCRM was defined as tumor lying within 1 mm of the mesorectal fascia; (9) MRI-detected T (mrT) stage: T1 stage, tumor signal intensity is limited to the submucosal layer. T2 stage, tumor signal intensity extends the mucosal layer but does not exceed the muscular layer. T3 stage, tumor signal intensity grows through the muscle layer and penetrates into the mesorectal fat of the rectum. T4 stage, tumor signal intensity infiltrates the visceral peritoneum and/or invades the adjacent organs. (10) MRI-detected lymph node (mrN) stage: N0 (no positive lymph node), N1 (1–3 positive lymph node) or N2 (≥ 4 positive lymph node). It was considered positive if lymph node showed suspicious morphology (round shape, irregular border, heterogeneous signal) and short axis diameter > 9 mm [13]; (11) enhancement pattern (EP): Mild/Moderate or obvious enhancement of tumor compared to normal rectal wall enhancement (Fig. 2a,b); and (12) rM: presence of metastasis determined according to radiological examination. Discrepancies between the radiologists were resolved by consensus after joint re-evaluation of the images and confirmed by another radiologist with 20 years of experience in abdominal diagnosis.

### Pathological analysis

The parenchymal parts of the surgically removed tumor were stained with regular hematoxylin-eosin (HE) and IHC marker of Ki-67. The interpretation of Ki-67 utilizes visual assessment under light microscopy. Ki-67 positive cell expression were indicated by brown-yellow or brown after staining. After browsing the entire pathological slice, positive cells were counted and the ratio of positive cells were calculated. The Ki-67 proliferation index was calculated based on the percentage of Ki-67 positive cells in the total carcinoma cells (reported at 10% intervals as 10%, 20%, 30%, etc.). 50% was used as the critical value to divide Ki-67 expression into low expression (< 50%) and high expression (≥ 50%) groups [27, 28]. Ki-67 expression in rectal cancer was tested by 2 clinical physiologists (with 10 and 15 years of work experience). Disagreement of the outcome was resolved by discussion and confirmed by a third clinical physiologist (with 22 years of work experience).

### Image segmentation and feature extraction

Lesion extraction was based on T2WI, DWI, and contrast-enhanced T1WI (CE- T1) sequences of MRI to plot the ROI and the image was output in a DICOM format. The ROIs were created manually via the open-source ITK-SNAP 3.4.0. A radiologist with 8 years of experience in abdominal imaging diagnosis determined the contours and plotted the shape along the boundaries by layers. Another radiologist with 15 years of abdominal imaging diagnosis then calibrated and verified the results before the entire tumor scope was plotted and a 3D-Mask was generated. To avoid data heterogeneity, all the DICOM images were subjected to normalization and resampled to the same resolution (1 mm×1 mm×1 mm). An extension software package in Python, pyradiomics, was used for feature extraction. DWI (maximum b value) was used as the original image, the plotted 3D-Mask was copied to an ADC map for feature extraction. Z-score standardization was adopted to downscale the dimension of each feature to the same order of magnitude before feature extraction. The selected features were further handled by using Max-Relevance and Min-Redundancy (mRMR) and recursive feature elimination methods. Then, the least absolute shrinkage and selection order (LASSO) linear regression method was used to reduce feature dimensions to identify features with highest correlation for radscore model construction.

### Model construction and validation

**Fig. 3:**
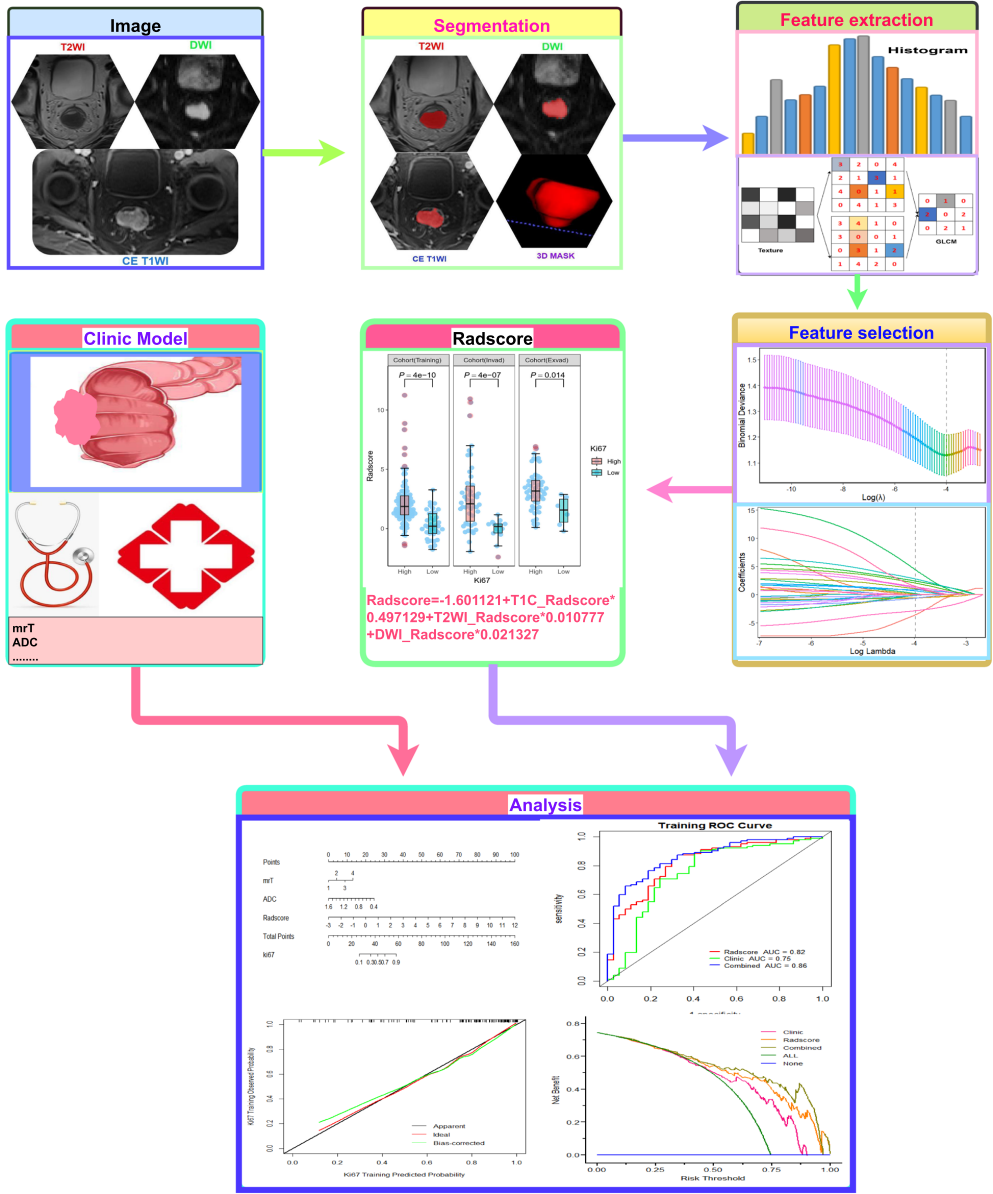
Workflow of multi-parametric MRI radiomics for predicting Ki-67 expression in rectal cancer

Each potential clinical risk factor and radiomics marker in the training cohort were analyzed through univariate and multivariate logistic regression analysis to screen independent predictors of Ki-67 expression in rectal cancer to construct the prediction models. Ki-67 was used as the dependent variable, radscore and clinical information were used as the independent variables to calculate the regression coefficients. Through weighted linear combination, a combined model was constructed and nomograms were generated. Decision curve analysis (DCA) was adopted to evaluate the clinical utilities. Calibration curves were used to assess the consistency between the model-predicted probability and the actual probability for Ki-67 expression (Fig. 3).

### Statistical analysis

The R software (version 3.6.1, http://www.r-project.rog) was used for statistical analysis. Normally distributed continuous variables were represented as means ± standard deviation. Categorical variables were represented by frequency and percentage. The caret software package was used to segment the queue and preprocess features. A confusion matrix was established to obtain accuracy, sensitivity, and specificity data. Multivariate logistic regression analysis was performed to select clinical features. ROCs were drawn to evaluate the model’s prediction performance via AUC, accuracy, and 95% CI (confidence interval). ROCs of each prediction model were compared and validated using Delong’s test. The rms and rmda software packages in R were used for calibration curve analysis and DCA, respectively. Two-tailed test results with *p* < 0.05 indicated statistical significance.

## Results

### Comparison of clinical and image features between high and low Ki-67 expression groups in patients with rectal cancer

Clinical and imaging data in two centers were summarized in Table 2. In the three cohorts, the age, sex, CEA expression, neoadjuvant chemotherapy, location, long diameter and rM stage of the patients were not statistically different between the high and low Ki-67 expression groups (*p* > 0.05). In contrast, the ADC value and mrT stage differed significantly between patients with high and low Ki-67 expression (*p* < 0.05). In the training and in-valid cohorts, mrN stage was significantly different between patients with high and low Ki-67 expression (*p* < 0.05); however, no significant difference was observed for the ex-valid cohort (*p* > 0.05). In the training cohort, CIR differences were significant between patients with high and low Ki-67 expression (*p* < 0.05); however, no significant difference was observed in the other two cohorts. In the training and ex-valid cohorts, mrCRM was significantly different (*p* < 0.05); whereas no significant difference was observed in in-valid cohort (Table 3).

**Table 2:** Clinical and imaging data in two centers

Characteristic	Center 1 (*n* = 199)	Center 2 (*n* = 60)
Ki67(H,%)	148 (74.37%)	50 (83.33%)
Age(y)	63.82 ± 11.38	68.45 ± 9.76
Sex(F,%)	75 (37.69%)	21 (35%)
CEA(Positive,%)	94 (47.24%)	23 (38.33%)
NC(Yes,%)	42 (31.66%)	17 (28.3%)
Location(Middle,%)	57 (41.71%)	31 (51.67%)
LD(mm)	42.2 (27.8)	48.3 (33.2)
ID(mm)	14.0 (8.5)	15.55 (5.5)
CIR(%)	75.0 (25.0)	84.83 (33.0)
ADC value(×10^−3^mm^2^/s)	0.81 ± 0.17	0.8 ± 0.11
EP(Obvious,%)	106 (53.27%)	32 (53.33%)
mrT stage(T3-4,%)	128 (64.32%)	32 (53.33%)
mrN stage(N0,%)	115 (57.79%)	36 (60%)
rM stage(Positive,%)	16 (8.04%)	6 (10%)
mrCRM(Positive,%)	59 (29.65%)	24 (40%)

Continuous variables (LD, ID, CIR ) are presented as the median and interquartile range (IQR)

*ADC* apparent diffusion coefficient, *CEA* carcinoembryonic antigen, *CIR* circum-involvement ratio, *EP* Enhancement pattern, *ID* Infiltration depth, *LD* Long diameter, *mrCRM* circumferential resection margin of MR, *mrT stage* T stage of MR, *mrN stage* N stage of MR, *NC* Neoadjuvant chemotherapy, *rM* assess metastasis by radiological examinations

**Table 3:** Comparison of Clinical and imaging characteristics with different Ki67 status in three cohorts

Characteristic	Training		*P1*	In-valid		*P2*	Ex-valid		*P3*
	Ki67 (L)	Ki67 (H)		Ki67 (L)	Ki67 (H)		Ki67 (L)	Ki67 (H)	
	(*n* = 37)	(*n* = 102)		(*n* = 14)	(*n* = 46)		(*n* = 10)	(n = 50)	
Age(y)	63.27 ± 12.25	64.02 ± 11.1	0.733	66.29 ± 12.41	63.46 ± 13.64	0.491	67.6 ± 11.71	68.62 ± 9.45	0.766
Sex			0.695			0.751			0.47
F	13	41		4	17		2	19	
M	24	61		10	29		8	31	
CEA			0.562			0.759			0.485
Negative	14	46		7	19		5	32	
Positive	23	56		7	27		5	18	
NC			0.096			0.777			0.709
Yes	7	35		4	17		2	15	
No	30	67		10	29		8	35	
Location			0.568			0.135			0.687
Upper	5	21		5	8		3	13	
Middle	15	42		3	23		4	27	
Low	17	39		6	15		3	10	
LD(mm)	44.0(33.35)	42.6(24.5)	0.746	42.2(42.5)	45.0(30.0)	0.975	43.5(39.5)	49.7(33.2)	0.974
ID(mm)	14.7(6.5)	16.3(5.15)	0.376	12.4(6.8)	19.0(7.5)	0.304	13.2(4.6)	16.0(6.35)	0.008
CIR(%)	68.5(42.5)	90.0(50.0)	0.021	75.0(50.0)	80.0(50.0)	0.809	84.1(33.0)	85.0(33.1)	0.861
ADC value	0.89 ± 0.20	0.78 ± 0.17	0.001	0.87 ± 0.94	0.77 ± 0.13	0.01	0.86 ± 0.09	0.78 ± 0.1	0.01
EP			0.702			0.759			1
Mild/Moderate	20	50		6	17		5	23	
Obvious	17	52		8	29		5	27	
mrT stage			0.001			0.036			0.003
T1	10	6		2	2		4	2	
T2	13	23		5	10		4	18	
T3	8	49		7	16		2	22	
T4	6	24		0	18		0	8	
mrN stage			0.038			0.015			0.362
N0	27	62		11	16		8	28	
N1	2	25		2	17		1	13	
N2	8	15		1	13		1	9	
rM stage			0.112			1			0.577
Negative	37	93		13	40		10	44	
Positive	0	9		1	6		0	6	
mrCRM			0.043			0.154			0.04
Negative	30	64		13	33		9	27	
Positive	7	38		1	13		1	23	

Sex, CEA, Neoadjuvant chemotherapy, tumor location, EP, mrT stage, mrN stage, rM stage, mrCRM status between two group was compared by Chi-square test. After homogeneity test, T-test was used to compared with Age, and ADC values. Man-Whitney U test was used to compared with CIR, LD, ID

*In-valid* Internal validation, *Ex-valid* External validation, *EP* Enhancement pattern

### Radiomics characteristics

In the training cohort, image features from T2WI, DWI, and CE-T1 were selected after consistency evaluation. A total of 2553 features were initially extracted. Then mRMR and LASSO were used for dimensionality reduction to select 18 features with strongest correlation (5 for T2WI, 8 for DWI, and 5 for CE-T1) (Table 4). Logistic regression was used to calculate the regression coefficient of radscore on the dependent variable Ki-67 in DWI, T2WI, and CE-T1, estimate the radiomics marker radscore, and construct a radscore model (Radscore=-1.601121 + CE-T1_Radscore*0.497129 + T2WI_Radscore*0.010777 + DWI_Radscore*0.021327).

**Table 4:** The selected radiomics features (5 for T2WI, 8 for DWI, and 5 for CE-T1) and their relevant coefficients in DWI-score, T2-score and CE-T1 were shown respectively

Sequence	Feature	Regression coefficient
DWI	wavelet-LHL_glcm_Correlation	-0.1513774
	original_shape_Flatness	-0.3334344
	wavelet-HLL_firstorder_Mean	1.3887476
	wavelet-LLL_firstorder_Kurtosis	0.7470276
	wavelet-HHH_gldm_SmallDependenceHighGrayLevelEmphasis	0.6402795
	wavelet-HHL_glcm_ClusterShade	0.9630735
	original_shape_Sphericity	-0.2369538
	wavelet-HHL_firstorder_Median	1.1780915
T2WI	original_firstorder_Minimum	-0.4911958
	wavelet.HHH_firstorder_Skewness	1.5153604
	wavelet.HHL_glszm_SmallAreaEmphasis	0.160303
	wavelet.LLL_glcm_Idmn	0.9831785
	wavelet.HLL_firstorder_Skewness	-1.2942632
CE-T1	wavelet.HHH_glszm_SizeZoneNonUniformity	0.04780355
	wavelet.HHH_gldm_DependenceVariance	0.19039054
	wavelet.HLL_firstorder_Skewness	0.66347437
	wavelet.HHH_glszm_SmallAreaLowGrayLevelEmphasis	-0.76497997
	original_shape_Sphericity	-1.64123212

DWI Radscore=-0.4578609+wavelet-LHL_glcm_Correlation*-0.1513774 +original_shape_Flatness*-0.3334344 +wavelet-HLL_firstorder_Mean*1.3887476+wavelet-LLL_firstorder_Kurtosis*0.7470276+wavelet-HHH_gldm_SmallDependenceHighGrayLevelEmphasis*0.6402795+ wavelet-HHL_glcm_ClusterShade *0.9630735+ original_shape_Sphericity*-0.2369538+wavelet-HHL_firstorder_Median *1.1780915

T2WI_Radscore=0.2927850+original_firstorder_Minimum*-0.4911958 +wavelet.HHH_firstorder_Skewness*1.5153604+wavelet.HHL_glszm_SmallAreaEmphasis *0.1603030+wavelet.LLL_glcm_Idmn*0.9831785+wavelet.HLL_firstorder_Skewness*-1.2942632

CE-T1_Radscore=1.80497344+wavelet.HHH_glszm_SizeZoneNonUniformity *0.04780355+wavelet.HHH_gldm_DependenceVariance *0.19039054+wavelet.HLL_firstorder_Skewness *0.66347437+wavelet.HHH_glszm_SmallAreaLowGrayLevelEmphasis*-0.76497997+original_shape_Sphericity* -1.64123212

### Features for Model construction

**Fig. 4:**
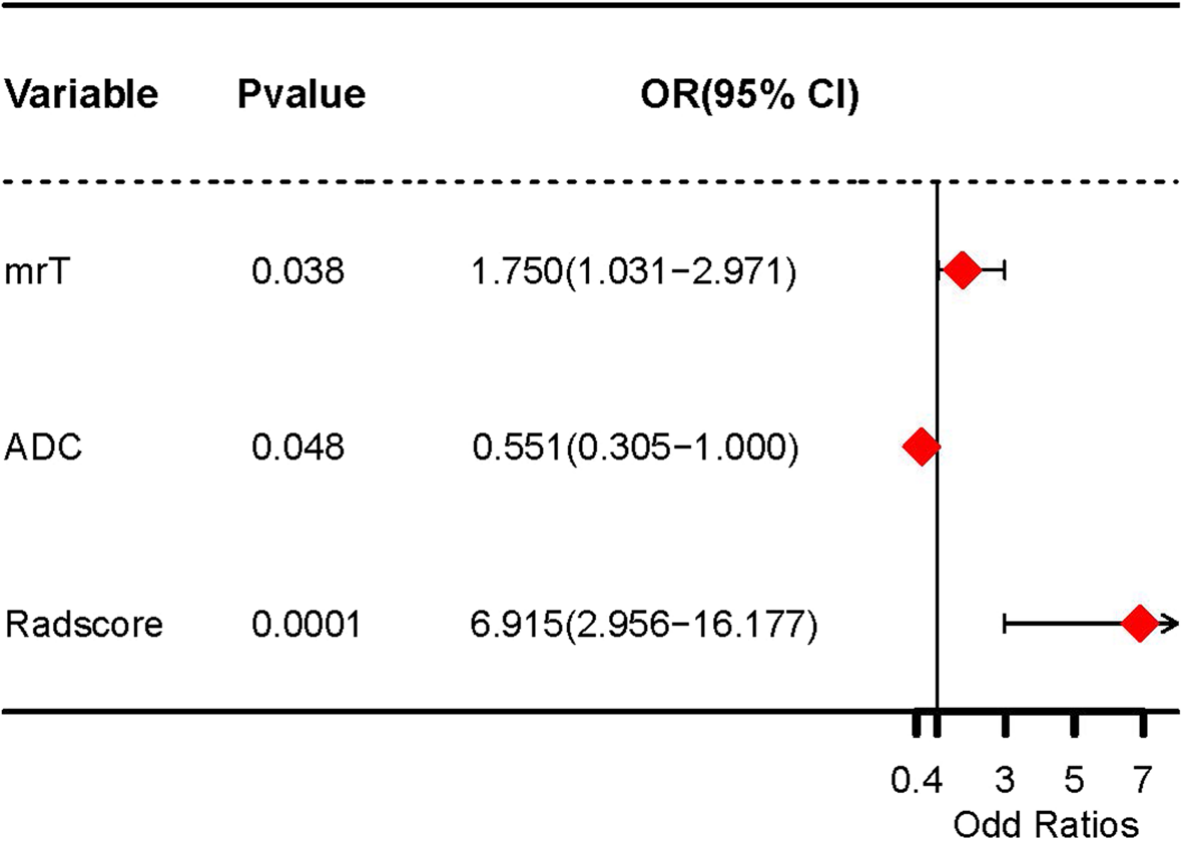
COX regression forest of the independent factors for predicting Ki-67 expression in rectal cancer

In the training cohort, features with significant differences namely Radscore, CIR, ADC value, mrT stage, mrN stage and mrCRM were analyzed using multivariate logistic regression. Radscore, mrT stage and ADC value were identified as the independent factors for predicting Ki-67 expression in rectal cancer (Table 5; Fig. 4). Then mrT stage and ADC value were used to construct a clinical model. Finally, the clinical and radscore model were combined to construct a combined model.

**Table 5:** Univariate and multivariate analyses of factors for assessing the status of Ki67

Risk factors	Univariate Analysis			Multivariate Analysis		
	OR	95%CI	*P* value	OR	95%CI	*P* value
CIR	0.391	0.038–4.042	0.431			
ADC value	0.042	0.002–0.774	0.026	0.55	0.30-1.0	0.048
mrCRM	1.009	0.272–3.742	0.11			
mrT stage	1.716	0.87–3.39	0.011	1.75	1.03–2.97	0.038
mrN stage	0.505	0.218–1.172	0.112			
Radscore	2.722	1.714–4.321	< 0.001	6.92	2.96–16.18	< 0.001

### ROC analysis

**Fig. 5:**
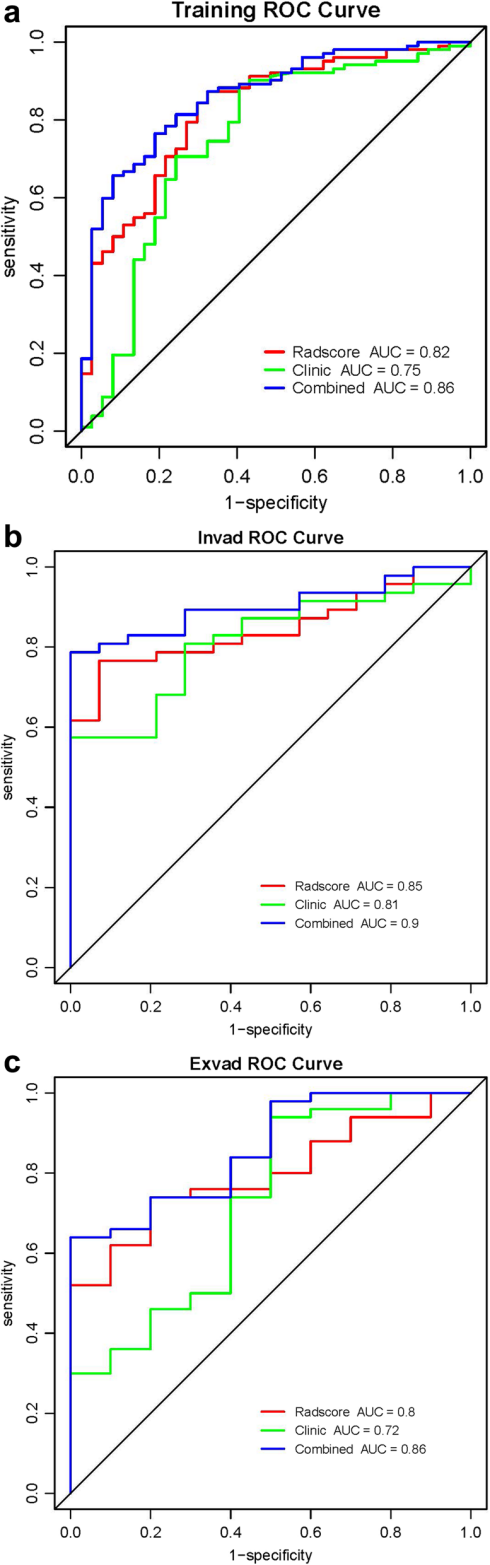
ROC analysis of the prediction model in the training cohort (**a**), in-valid cohort (**b**), and ex-valid cohort (**c**)

For all cohorts, AUC was consistently higher in the combined model, followed by the radscore model and then the clinical model (Fig. 5a-c). For the radscore model, AUCs in the training, in-valid, and ex-valid cohorts were 0.81, 0.83, and 0.78, respectively. While for the combined model, AUCs were 0.84, 0.88, and 0.85 for the three cohorts. Accuracy, AUC, Sensitivity, Specificity, Positive predictive value (PPV) and Negative predictive value(NPV) for all models are presented in Table 6. Delong tests were conducted for all models among the three cohorts (Table 7). In the three cohorts, the prediction performance of the combined model for Ki-67 expression was greater than that of the clinical model (*p* < 0.05), and no significant difference was observed between Radscore model and combined model (*p* > 0.05).

**Table 6:** The ROC analysis of the different models

Cohort	Model	Accuracy	AUC(95%CI)	Sensitivity	Specificity	PPV	NPV
Training	Radscore	0.81	0.83 (0.747–0.903)	0.83	0.76	0.90	0.62
	Clinical	0.74	0.75 (0.646–0.853)	0.76	0.68	0.87	0.51
	Combined	0.84	0.86 (0.794–0.928)	0.84	0.73	0.90	0.61
In-valid	Radscore	0.83	0.85 (0.751–0.942)	0.85	0.79	0.93	0.61
	Clinical	0.80	0.81 (0.702–0.924)	0.80	0.79	0.93	0.55
	Combined	0.88	0.90 (0.826–0.976)	0.87	0.93	0.98	0.68
Ex-valid	Radscore	0.78	0.80 (0.673–0.919)	0.78	0.79	0.92	0.52
	Clinical	0.72	0.72 (0.533–0.911)	0.74	0.64	0.87	0.43
	Combined	0.85	0.86 (0.749–0.971)	0.85	0.86	0.95	0.63

**Table 7:** Delong test for different models in three groups

Model	Training	In-valid	Ex-valid
Radscore vs. Clinical	0.261	0.616	0.505
Radscore vs. Combined	0.163	0.076	0.228
Combined vs. Clinical	0.015	0.047	0.045

### Clinical application of nomograms

**Fig. 6:**
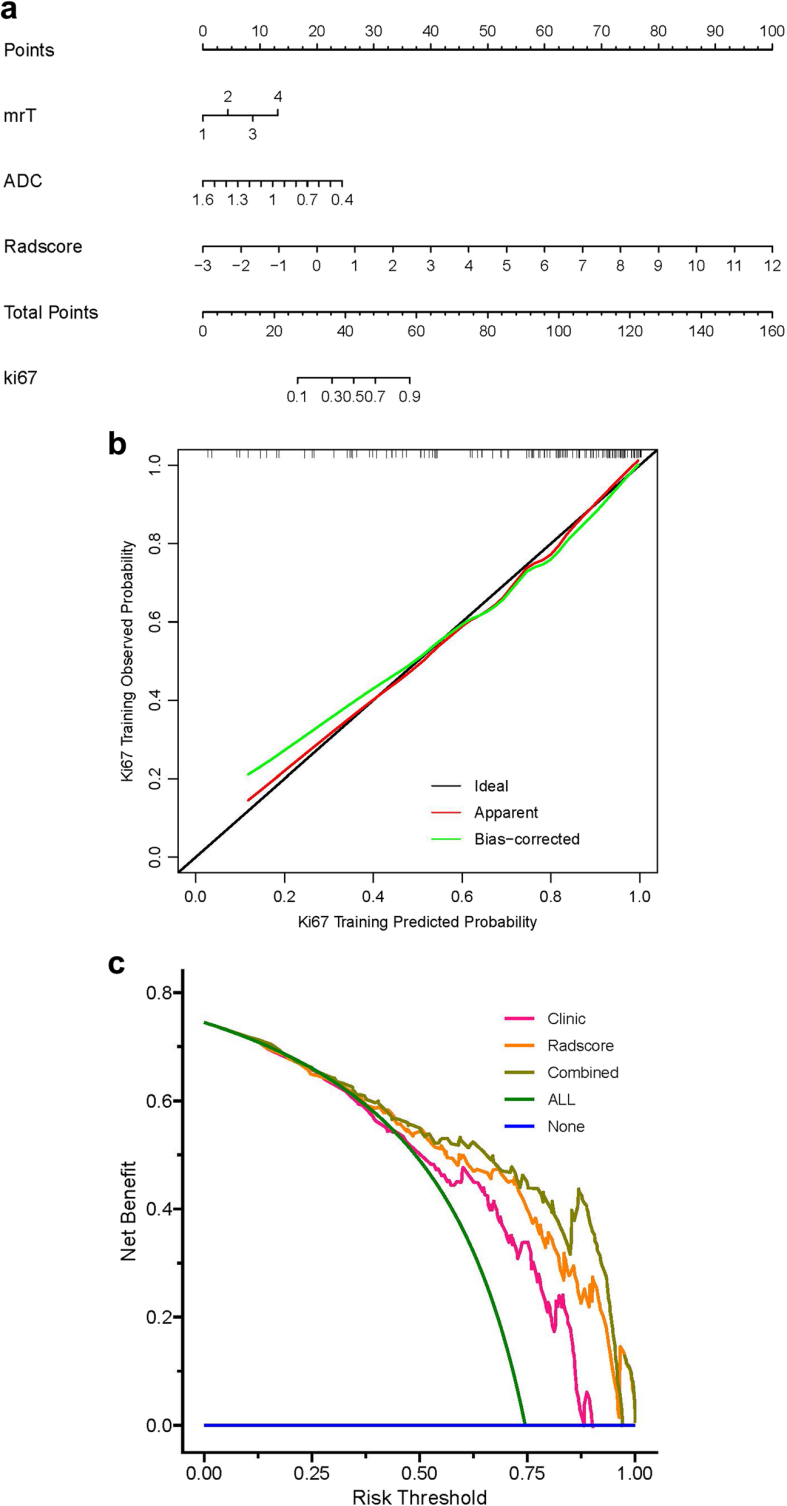
The nomogram of the combined model, which generated corresponding evaluation scores according to the respective contributions of the radiomics marker value, mrT staging, and ADC value to Ki-67 expression in the regression model. The score for each factor was summed to obtain the total score for the probability of predicting Ki-67 expression (**a**). Calibration curves of the nomogram. Calibration curves with slope near 1 indicate good fit and accurate nomograms (**b**). DCA of the nomogram, radiomics model and clinical model. Nomogram had superior capabilities for determining Ki-67 expression in rectal cancer. The y- and x-axes indicate net benefits and threshold probability, respectively (**c**)

The combined model was used to construct the nomogram. The nomogram calibration curves indicate that prediction outcomes of the training and validation datasets for Ki-67 expression in rectal cancer highly consistented with the postoperative pathological IHC results. DCA revealed that the nomogram based on the combined model had relatively good clinical performance (Fig. 6a-c).

## Discussion

In this study, multi-parametric MRI radiomics were used to conduct preoperative evaluation of Ki-67 expression in rectal cancer. The patients were divided into three cohorts (training, in-valid, and ex-valid cohorts). For each cohort, three prediction models (namely, radscore, clinical, and combined models) were established to predict Ki-67 expression in rectal cancer. Among the three cohorts, the combined model had a higher AUC than that of the other two models. The combined model had the highest accuracy for preoperative prediction of Ki-67 expression. The nomograms constructed based on the combined model could be served as intuitive and easy-to-use prediction tools for clinicians. Individualized prediction information was obtained through the simple scores provided, which facilitates their clinical decision-making and improves the prognosis of patients with rectal cancer.

Previous researches [27, 28] indicated that high Ki-67 expression (≥ 50%) in patients with colorectal cancer contributed to poor tumor differentiation and high metastatic recurrence risks. So Ki-67 expression is an independent prediction factor of poor prognosis of colorectal cancer, with 50% Ki-67 expression defined as the critical value [10, 27, 28]. Currently, Ki-67 expression of rectal cancer was generally obtained through invasive biopsy or surgical pathology tissues [29].

This study extracted features from three preoperative MRI sequences of patients with rectal cancer in the training cohort. Then 18 features significantly correlated with Ki-67 expression in rectal cancer were selected to construct models, among which wavelet features account for the majority (14/18, 77.8%), followed by original_shape features (3/18, 16.6%) and original_firstorder features (1/18, 5.6%). Other studies [30, 31] also showed that “wavelet” features had powerful prognostic abilities and were major components in building radiomic model or signature, which is consistent with our study. “Wavelet” features [30, 32], which are derived from the wavelet transform algorithm, can describe the texture information of images at different scales and provide valuable feature information for discrimination and classification of lesions that cannot be identified by the naked eye. The “original_shape” feature is used to describe the geometric features of the image, providing quantifiable indicators for tumor morphological analysis. “Original_firstorder” features are used to describe the distribution of gray values within an image for the discrimination and classification of lesions. These features not only represented the morphological characteristics but also indicated the heterogeneities of tumors that are correlated with tumor proliferation and prognosis.

Through a further fitting of the three sequences, we constructed a radscore model, which yielded AUCs in the training, in-valid, and ex-valid cohorts of 0.81, 0.83, and 0.78, respectively. Previous studies [33, 34] have suggested that models built based on multiple sequences outperformed those based on a single sequence in evaluation of extramural venous invasion status, T staging and neoadjuvant chemotherapy outcomes for rectal cancer. Shu et al. [33] used multi-sequence MRI to select 20 features for construction of a radscore model that successfully evaluated EMVI in rectal cancer. The AUC for the training and validation cohorts were 0.744 and 0.738, respectively. You et al. [34] used T2WI and ADC map to construct a radscore model to evaluate T staging of rectal cancer; the model’s AUCs were higher than those obtained through single sequences. However, these radscore models based on multiple sequences lacked external validation to confirm the model stability in evaluation of postoperative pathological status of rectal cancer. By contrast, our study included an internal validation cohort and an external validation cohort to demonstrate the reliability and stability of the constructed model.

In the training cohort, patient clinical information and preoperative MRI examinations were evaluated for feature extraction to construct a clinical model. ADC value and mrT stage revealed by a multivariate regression analysis to be independent predictors of Ki-67 expression in rectal cancer and were thus used to establish a clinical model. Previous reports [35, 36] showed accurate clinical staging of rectal cancer was closely correlated with the selection of individualized treatment plans and prognosis. Different stages of rectal cancer require correspondingly individualized treatment methods, including resection surgery, chemotherapy, neoadjuvant chemotherapy, or neoadjuvant radiotherapy [36]. DWI, which reflects tumor cell density and necrosis, is the only noninvasive method for detecting water molecule diffusion in living tissues, while the ADC value is a quantitative indicator of DWI [37]. Many studies have indicated that the ADC value is useful for predicting rectal cancer prognosis [37, 38]. A study by our team [1] analyzed the correlation between T staging and ADC in 77 patients with rectal cancer, which revealed that Ki-67 expression in rectal cancer is negatively correlated with ADC. Higher Ki-67 expression corresponds with lower ADC value. Consistently, the pathological T (pT) staging of rectal cancer is negatively correlated with ADC. The higher the pT staging, the lower the ADC value. A study [37] investigated 91 cases of patients with rectal cancer and reported that the ADC value was positively correlated with histological differentiation but negatively correlated with Ki-67 expression. According to the 8th AJCC stratification system, the anatomic extent (T stage) is one of the most important prognostic factors for primary colorectal cancer. The 5-year disease-free survival (DFS) and 5-year overall survival (OS) were different among patients with different T stages. The higher the T stage, the lower the 5-year DFS and 5-year OS. It should be noted that other factors also contribute to the prognosis of patients, such as tumor differentiation, lymph node metastasis, lymphovascular invasion, perineural invasion, etc. [39, 40]. Hence, these factors should be comprehensively considered before making an individualized treatment plan.

The present study revealed that the combined model successfully predicted Ki-67 expression levels in the training, in-valid, and ex-valid cohorts. The AUC value of the combined model was higher than that of the other models. Delong test results revealed that the prediction ability of the combined model was superior to the clinical model for all cohorts. However, the performance of the combined model and the radscore model were not significantly different across all three cohorts. The nomogram revealed that higher radscore values, deeper tumor infiltration, and lower ADC values were correlated with higher Ki-67 expression. We further used DCA to quantify net benefits of nomogram for individualized prediction under different threshold probabilities, which revealed that the net benefits of nomogram-predicted Ki-67 expression outperformed those of the clinical model and radscore model. Cai et al. [5] collected 149 patients with rectal cancer and plotted ROIs on T2WI, DWI, CE-T1 and ADC maps, respectively. Then, they were employed to screen features and construct a radiomics signature for predicting tumor-stroma ratio in rectal cancer. Both mean ADC and rad-score showed a positive correlation with the tumor-stroma ratio in the training group. However, the AUCs of the rad-score were better than that of the mean ADC in the training and validation groups. Although the model achieved good predictive performance, it lacked external validation to determine whether it was suitable for data from other centers. Meng et al. [30] used multi-parametric MRI data to construct radiomic models for predicting multiple biological characteristics (Ki67 expression, lymph node metastasis, tumor differentiation, HER-2 and KRAS-2 mutation) of rectal cancer, with AUC values ranged from 0.651 to 0.699. Compared with these studies [5, 30], our model had the following advantages: (1) The model achieved satisfactory prediction performance verified both in the internal and external validations, implying that it was stable and reliable. (2) After adding clinical information, the predictive performance of the combined model was improved. Hence the Nomogram based on the combined model can be served as an easy-to-use tool for Ki-67 prediction in patients with rectal cancer and has potential for clinical applications.

The present study had the following limitations. (1) Retrospective data from patients diagnosed with rectal cancer through surgery were collected for analysis, resulting in selection bias. (2) Few patients with rectal cancer had low Ki-67 expression. This may explain the poor prognosis of most rectal cancer cases and requires more data for validation. (3) In spite of limited training samples, the predictive performance of the model constructed in present study was satisfactory. We argue that more data in the training cohort may further improved the performance of the predicting radiomics model. Therefore, a larger sample will be collected in future studies.

## Conclusions

In conclusion, we constructed a multi-parametric MRI radiomics model for preoperative prediction of Ki-67 expression in patients with rectal cancer. The proposed prediction model had superior performance and was validated in two centers. The model was stable and reliable by internal and external validations. The combined model had the best prediction performance, thus the nomograms constructed based on the combined model could provide doctors with a noninvasive and accurate preoperative tool to support clinical decision-making.

## Data Availability

All data generated or analyzed throughout this study are included in this published article.

## Abbreviations

ADC: Apparent diffusion coefficient
AUC: Area under the curve
CEA: Carcinoembryonic antigen
CE-T1: Bontrast enhancement T1WI
CI: Confidence interval
CIR: Circum-involvement ratio
DCA: Decision curve analysis
DWI: Diffusion-weighted imaging
EP: Enhancement pattern
Ex-valid: External validation
HE: Hematoxylin-eosin
in-valid: Internal validation
LASSO: Least absolute shrinkage and selection order
IHC: Immunohistochemistry
mrCRM: Circumferential resection margin status base on MRI
mrN: N staging of tumor based on MRI
mrT: T staging of tumor based on MRI
NPV: Negative predictive value
PPV: Positive predictive value
ROC: Receiver operating characteristic
ROI: Region of interest
rM: Metastasis according to radiological examination
mRMR: Max-relevance and min-redundancy
